# Family history, waist circumference and risk of ischemic stroke: A prospective cohort study among Chinese adults

**DOI:** 10.1016/j.numecd.2023.01.009

**Published:** 2023-01-18

**Authors:** Lei Liu, Xiaojia Xue, Hua Zhang, Xiaocao Tian, Yunhui Chen, Yu Guo, Pei Pei, Shaojie Wang, Haiping Duan, Ruqin Gao, Zengchang Pang, Zhengming Chen, Liming Li

**Affiliations:** a Department of Epidemiology and Health Statistics, School of Public Health, Qingdao University, Qingdao 266071, China; b Qingdao Municipal Center for Disease Control and Prevention, Qingdao Institute of Preventive Medicine, Qingdao 266033, China; c Department of Vascular Surgery, Qingdao Municipal Hospital, Qingdao 266071, China; d National Center for Cardiovascular Diseases, Fuwai Hospital Chinese Academy of Medical Sciences, Beijing 100037, China; e Chinese Academy of Medical Sciences, Beijing 102308, China; f Clinical Trial Service Unit and Epidemiological Studies Unit (CTSU), Nuffield Department of Population Health, Medical Research Council Population Health Research Unit, University of Oxford, Oxford OX3 7LF, United Kingdom; g Department of Epidemiology & Biostatistics, School of Public Health, Peking University, Beijing 100191, China; h Peking University Center for Public Health and Epidemic Preparedness & Response, Beijing 100191, China

**Keywords:** China, Family history, Waist circumference, Ischemic stroke, Prospective study

## Abstract

**Background and Aims:**

The associations between genetic factors and waist circumference (WC) with stroke risk have been evaluated in Western studies. However, evidence of this association has rarely been reported in the Chinese population. This study aimed to evaluate the association between WC and family history of stroke (FHS) with ischemic stroke (IS) risk among Chinese adults and to further explore the potential interaction of these associations.

**Methods and Results:**

The China Kadoorie Biobank (CKB) study recruited 35,508 participants aged 30–79 years from the Qingdao urban area during 2004–2008. A total of 33,355 participants were included in study. Cox regression analysis was used to estimate the multivariable-adjusted hazard ratios (HR) and 95% confidence intervals (CI) for the independent and interactional associations between FHS and WC and IS risk. Participants with FHS had a 29% (HR = 1.29, 95% CI: 1.12–1.50) higher IS risk than those without FHS. Participants with excessive WC (85 cm for males and 80 cm for females) had a 78% (HR = 1.78, 95% CI: 1.51–2.10) higher IS risk than those with normal WC. The combined effect of FHS and excessive WC on IS was statistically significant (HR= 2.29, 95% CI: 1.84–2.86). The present study further found statistically significant multiplicative interactions of FHS and WC with IS risk (*P*_interaction_ < 0.001).

**Conclusion:**

The present study indicated that FHS and WC were significantly associated with an increased risk of IS. The association between FHS and IS was associated with excessive WC.

## Introduction

Stroke is a prominent cause of mortality and disability, and an important contributor to the economic burden [1]. From 1990 to 2019, stroke has become the second-leading cause of death worldwide and the third-leading cause of death and disability combined [2]. Ischemic stroke (IS) is the most frequent among incident cases, constituting 87% of all strokes [3]. The incidence rate of IS in China is 36% higher than the global average [4]. As such, IS poses a major public health burden in China.

Epidemiological studies have demonstrated that stroke is a complex, multifactorial disease caused by a combination of vascular, environmental, and hereditary factors [5]. Large meta-analyses have found that family history of stroke (FHS) increases the stroke risk by 30–44% [6]. Family-history studies can be used to investigate the heritability of complex diseases and potential interactions between genetic and environmental factors [7]. Accumulating evidence suggests that, in a shared environment, individuals with FHS have genetic susceptibility to stroke when compared to individuals without FHS [8]. Parental history of stroke and sibling history of stroke may increase the likelihood of having a more severe stroke [8, 9]. The mean waist circumference (WC) and prevalence of abdominal obesity significantly increased among Chinese adults between 1993 and 2015 [10]. WC is a better tool for assessing body composition and visceral adiposity and reflects cardiometabolic profiles [11]. Abdominal obesity assessed by WC is more pathogenic and is thus more closely associated with cardiovascular diseases [12, 13]. However, cohort studies on the relationship between FHS, WC, and IS risk in the Chinese population are limited.

The present study aimed to evaluate the relationship of FHS and WC with IS risk in a large-scale, Chinese population-based cohort study. We further prospectively examined the interaction of FHS and WC with the risk of IS in Chinese males and females.

## Methods

### Study participants

The participants came from the China Kadoorie Biobank (CKB) study in Qingdao. More detailed information has been described previously [14–16]. In brief, a total of 35,508 urban residents aged 30–79 years were recruited between 2004-2008. All the participants then entered the follow-up period. Participants who self-reported cancer (n=162), coronary heart disease (n=1,827), or stroke (n=238) in the baseline survey were excluded, leaving 33,355 participants for the present study.

The study was approved by the Oxford Tropical Research Ethics Committee, University of Oxford (UK), the ethics review committee of the Chinese Center for Disease Control and Prevention (Beijing, China) and the ethics review committee of the Qingdao Municipal Center for Disease Control and Prevention (Shandong, China). All the participants provided written informed consent.

### Data collection

#### Baseline survey

A laptop-based questionnaire was administered by professionally trained interviewers as the baseline survey, including socio-demographic characteristics (age, sex, annual household income, education, occupation, and marital status), lifestyle (alcohol consumption, smoking status, diet frequency, physical activity, and menopause status only for women), personal and family medical history. Physical activity was quantified using the metabolic equivalent of tasks (METS) based on self-reported type, duration, intensity of physical activity, and time spent in sedentary activities.

All participants were asked whether their first-degree relatives (biological father, mother, or siblings) had been diagnosed with stroke. The number of siblings with stroke was recorded. We defined participants as “having a family history” when they reported at least one first-degree relative had stroke, and participants as “having parental history” when they reported that either the father, mother, or both had a stroke. Groups were always defined by the different types of familial history (paternal history, maternal history, parental history, siblings’ history).

Physical measurements for all participants were obtained, including height, weight, blood pressure, and WC. Participants wore light clothes and no shoes to measure height and weight, to the nearest 0.1 cm or 0.1 kg. Body mass index (BMI) was calculated as weight (kg) divided by the square of height (m^2^). Blood pressure was measured twice after the participants had remained at rest in a seated position for at least 5 mins. A third measurement was required if the blood pressure difference was more than 10 mmHg between the first two measurements, and the mean of the last two blood pressure values was used in the analyses.

WC was measured using a soft, non-stretchable tape (accurate to 0.1 cm), at the midpoint between the lowest rib and the iliac crest. We define excessive WC as ≥ 85 cm for males and ≥ 80 cm for females [17].

Spot random plasma glucose (RPG) was tested using the SureStep Plus System (Johnson&Johnson). RPG data were missing for 422 participants.

### Follow-up for IS

IS events were ascertained through linkage with established disease surveillance point systems (DSPs) and the national health insurance system [18], using the International Classification of Diseases, 10th Revision (I63). Follow-up time was calculated from the date of the baseline survey to the date of IS occurrence, loss to follow-up, or December 31, 2015, whichever occurred first.

### Statistical analysis

The baseline characteristics of the participants are presented as mean ± standard deviation or frequency (percentage, %). Cox proportional hazard regression models were used to examine the independent and multiplicative interactions of FHS and WC with IS risk, generating the hazard ratios (HRs) and 95% confidence intervals (CIs) and using the likelihood ratio test. The additive interaction effects were evaluated by the relative excess risk due to interaction (RERI), attributable proportion due to interaction (AP), and synergy index (SI) [19]. Potential non-linear associations between WC and IS risk were modelled using restricted cubic splines (RCS) modelling with WC as a continuous variable, with four degrees of freedom. For comparison purposes, HRs were extracted in the 5th, 25th, 75th, and 95th percentiles of the WC [20]. Subgroup analysis according to baseline characteristics was conducted to investigate the effects of these characteristics on the association between FHS or WC and IS risk.

Potential confounders were adjusted for using the following models: Model 1 was adjusted for sex (male or female) and age (continuous) at baseline (men and women were adjusted only for age). Model 2 was further adjusted for education (primary and below, junior high school, high school, and above), occupation (workers, administration/professional technology, sales and service staff/private, housework, retired, unemployed/laid off), annual household income (<20000, 20000–35000, ≥35000), marital status (yes or no), smoking status (never, current regular smoking), alcohol consumption (never, current regular drinking), diet frequency (red meat, fresh fruits, vegetables, and dairy products), physical activity (MET-hoday^-1^), menopausal status (only for women, no, pre-menopause, menopause), and FHS for the different WC groups. Participants who drank alcohol at least once a week or their equivalent for at least one year were defined as current regular drinkers. Participants who reported having smoked at least one cigarette daily or their equivalent for at least six months were defined as current regular smokers. Model 3 was further adjusted for systolic blood pressure (SBP, continuous), diastolic blood pressure (DBP, continuous), RPG (continuous), and BMI (continuous) in the different FHS groups.

If the 95% CI of RERI and AP did not include 0, and the 95% CI of SI did not include 1, the additive interactions were statistically significant. Statistical significance was indicated by a two-tailed *P*<0.05. All statistical analyses were conducted using SPSS 25.0 (Armonk, NY: IBM Corp), and the R Foundation for Statistical Computing (https://www.R-project.org/.).

## Results

### Baseline characteristics according to FHS and WC

The mean baseline age was 50.09 ± 9.88 years of 33,355 participants (14,908 for men, 18,447 for women). There were 5,227 (15.67 %) participants that had FHS, and 20,890 (62.63 %) participants that had excessive WC (⩾ 85 cm for males and ⩾ 80 cm for females). Compared to the participants with normal WC, those with excessive WC were more likely to be older, retired, have lower regular fresh fruit and dairy products intake, have lower physical activity, and have higher BMI, SBP, and RPG (*P* < 0.05) at baseline (Table 1).

### Associations of FHS and IS risk

Overall, 1,093 participants (3.28 %) were newly diagnosed with IS (530 men, 563 women) during 302,008.88 person-years (PYs) of follow-up. Per 1,000 PYs, the incidence rate of IS was 3.41 for participants without FHS, and 4.70 for participants with FHS (Table 2). Compared to the participants without FHS, those with FHS had a 29% higher risk of IS (HR=1.29, 95% CI: 1.12–1.50, Model 2), and the association remained with further adjustment for SBP, DBP, RPG, and BMI (Model 3). A positive association was observed in men (HR=1.55, 95% CI: 1.27–1.90), but was not observed in women (HR=1.06, 95% CI: 0.86–1.32) (Table 2). Compared to the participants without FHS, the adjusted HRs (95% CIs) were 1.33 (95% CI: 1.10–1.61), 1.28 (95% CI: 1.05–1.56), 1.27 (95% CI: 1.09–1.48), and 1.33 (95% CI: 0.96–1.85) for paternal, maternal, parental, and sibling history, respectively. A positive association was found in men, but not in women (Supplementary Figure 1). According to the number of FHS members with stroke, the adjusted HRs (95% CIs) were 1.23 (1.05–1.44) and 1.73 (1.24–2.40) for participants with FHS involving 1 and ⩾ 2 members, respectively, in comparison with those without FHS (Supplementary Table 1). The HRs increased with an increasing number of first-degree relatives with IS risk, and this trend remained after multivariable adjustment (*P*_trend_ <0.001) (Supplementary Table 1).

### Associations of WC and IS risk

**Figure 1:**
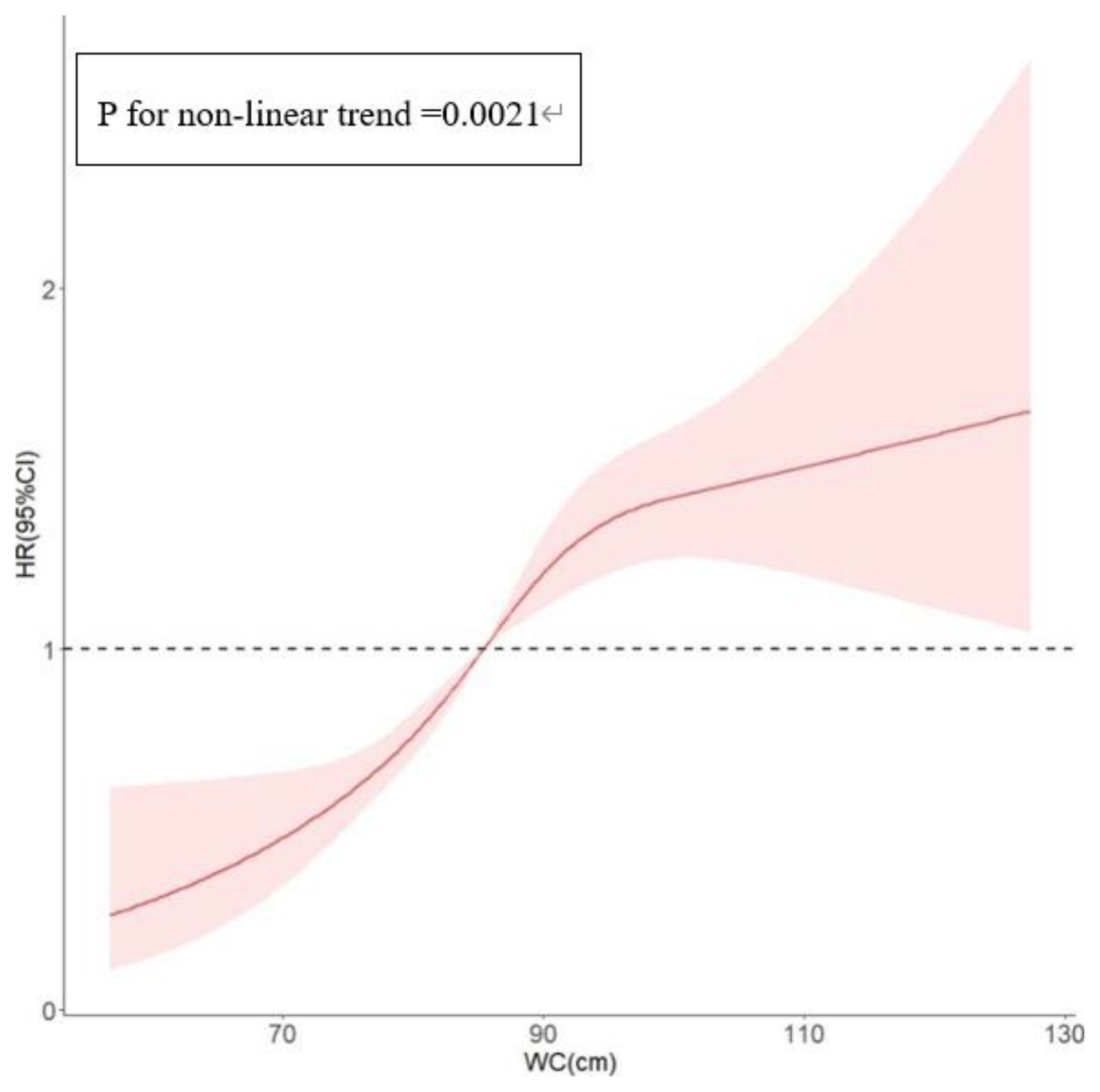
Plots of IS HR with 95% CI (shaded regions) from multivariate adjusted Cox regression analysis with the restricted cubic spline of waist circumference by adjusting of model 2. I WC, waist circumference; HR, hazard ratio; CI, confidence interval.

The incidence rate of IS was 1.60 for participants with normal WC, and 7.82 for participants with excessive WC per 1,000 PYs (Table 2). Compared to participants with the normal WC, excessive WC had a significant association with the risk of IS (HR=1.78, 95% CI: 1.51–2.10, Model 2). For men and women, the HRs were 1.72 (95% CI: 1.40–2.12) and 1.83 (95% CI: 1.40–2.39), respectively. The association remained after further adjustment for SBP, DBP, and RPG (Model 3) (Table 2). Incrementally higher risks of all four outcomes were observed in higher quartiles of IS risk for WC compared with the lowest quartile (Q1) group in all models (all *P*_trend_ < 0.001) (Supplementary Table 2). There was a tendency towards an S-shaped curve of WC with IS risk, and the association was significant (*P*_Non-linear_ = 0.002). The risk of IS increased with increasing WC (Fig. 1). Almost all plots of the IS risk hazard ratio showed wide confidence intervals among participants with FHS, especially for individuals at both ends of the WC measurement. Non-linear associations existed between WC and IS risk for parental history (*P*=0.0218) (Supplementary Figure 2).

### Interaction of FHS and WC with IS risk

**Figure 2:**
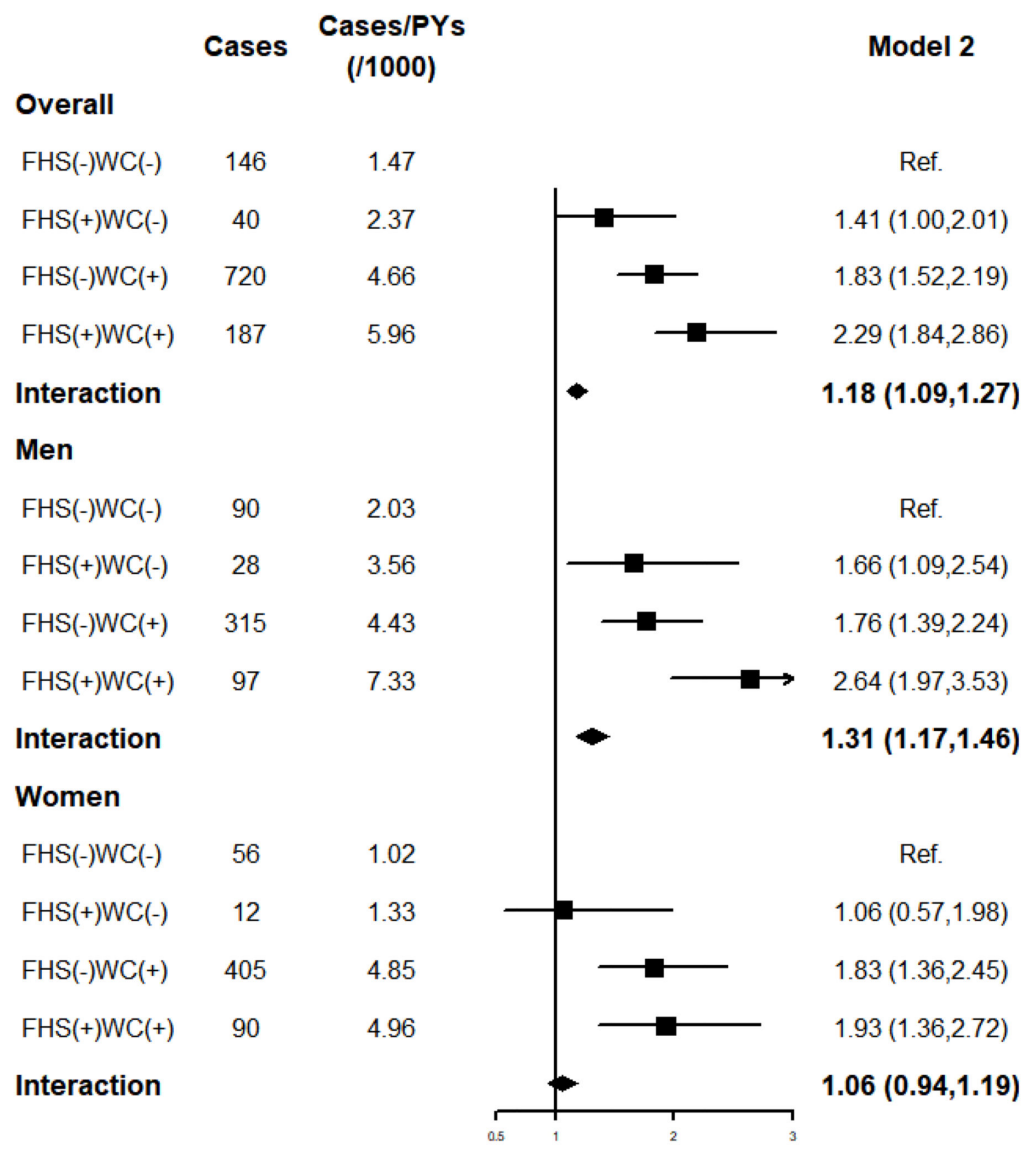
Interaction of family history and waist circumference on the risk of ischemic stroke. FHS (-), participants without FHS; FHS (+), participants with FHS; WC (-): normal WC; WC (+): excessive WC. FHS, family history of stroke; WC, waist circumference.

Multiplicative interactions between FHS and WC were statistically significant (*P* < 0.001). Compared to the participants with normal WC and without FHS, the adjusted HRs (95% CIs) for those with normal WC but with FHS, those with excessive WC but without FHS, and those with excessive WC and FHS were 1.41 (95% CI: 1.00–2.01), 1.83 (95% CI: 1.52–2.19), and 2.29 (95% CI: 1.84–2.86), respectively (Fig. 2). The multiplicative interaction between FHS and WC with IS risk was significant in men (*P* < 0.001), but not in women (*P* = 0.322) (Fig. 2). In the overall population, this study found a significant interaction between other types of family history, except for siblings. (Supplementary Figure 1). Additive interaction analysis after adjusting for relevant confounders showed no interaction between FHS and WC in the development of IS (Supplementary Table 3).

### Subgroup analyses

**Figure 3:**
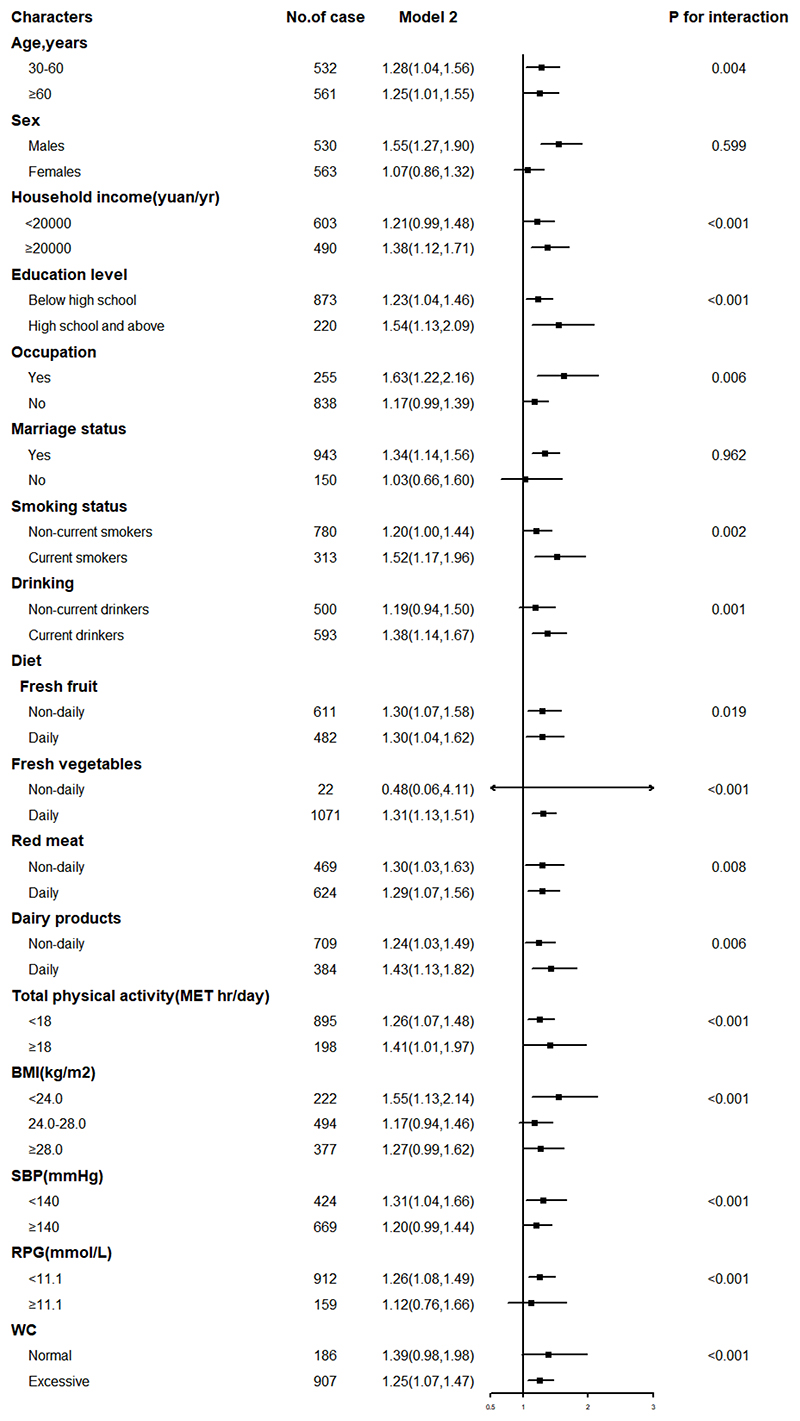
Subgroup analysis of associations between family history of stroke and risk of ischemic stroke according to potential baseline risk factors. MET, metabolic equivalent of task; BMI, body-mass index; SBP, systolic blood pressure; RPG, random plasma glucose; WC, waist circumference.

No significant interactions were found across the strata in terms of sex and marital status. However, the strength of the association between FHS and IS was largely consistent across subgroups defined by age, household income, education level, occupation, smoking status, drinking, frequency of fresh fruits, fresh vegetables, red meat, dairy products, physical activity, BMI, SBP, _and_ RPG (*P*_interaction_ < 0.05) (Fig. 3).

**Figure 4:**
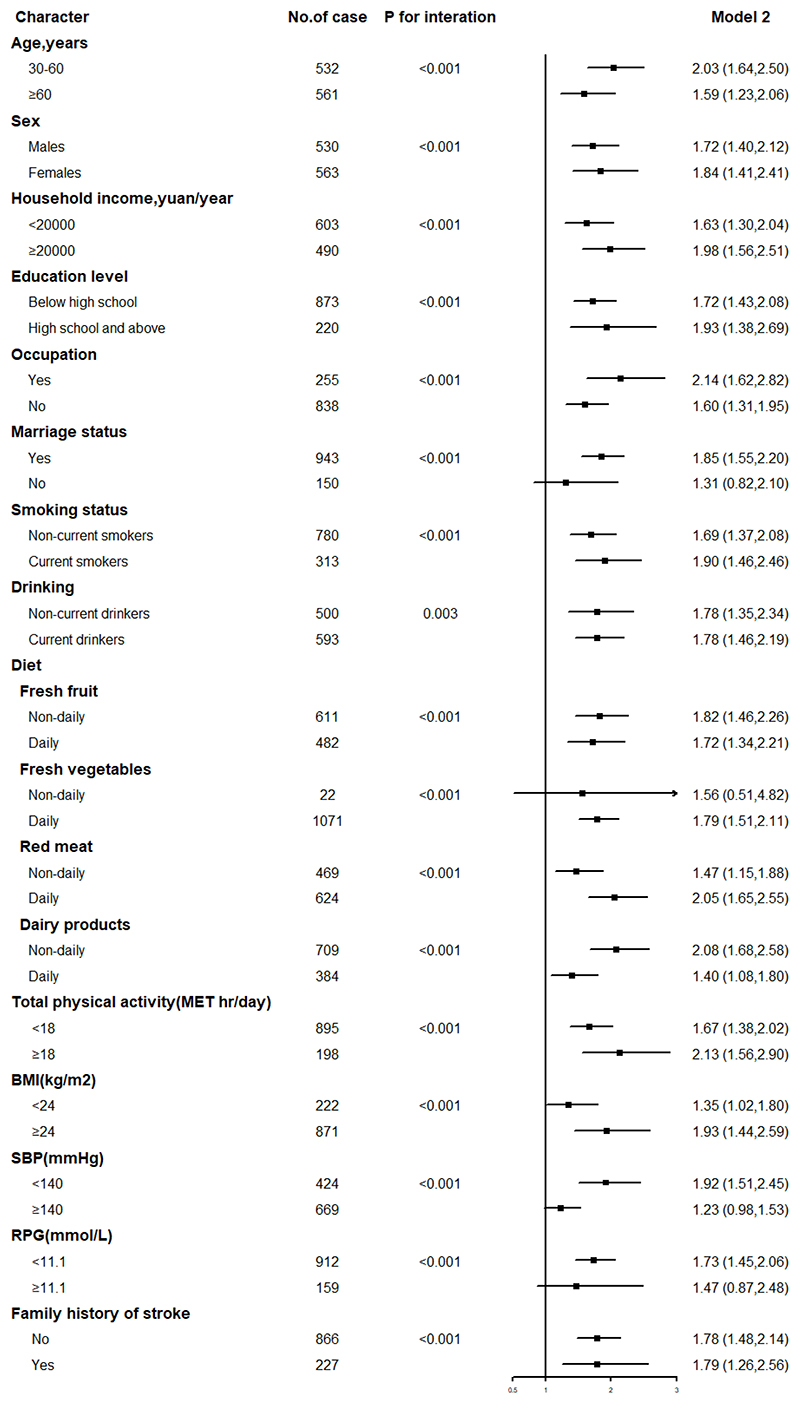
Subgroup analysis of associations between waist circumference and risk of ischemic stroke according to potential baseline risk factors. MET, metabolic equivalent of task; BMI, body-mass index; SBP, systolic blood pressure; RPG, random plasma glucose.

Significant differences in the association of WC and IS risk across age, sex, household income, education level, occupation, marital status, smoking status, drinking, the frequency of fresh fruits, fresh vegetables, red meat, dairy products, physical activity, BMI, SBP, and RPG were also observed in the present study (*P*_interaction_ < 0.05) (Fig. 4).

## Discussion

In this population-based prospective cohort study of Chinese adults with 9.05 years of follow-up, we found that either FHS or WC was positively associated with IS risk. In further analyses that combined the effects of FHS and WC, we observed that the FHS and abdominal adiposity defined by WC had a significant multiplicative interaction on IS risk, but no additive interaction. Participants who had FHS and excessive WC had a 129 % (164 % among men, 93 % among women) higher risk of IS compared with those without FHS and normal WC, which implied that excessive WC increased the risk of IS among people with FHS. FHS and excessive WC were associated with the greatest risk of IS. Our study revealed that young age was more strongly associated with FHS in comparison with old age. Moreover, FHS and WC interacted with health risk behaviors (smoking status and alcohol consumption), impacting the risk of IS occurrence.

## The association of FHS and IS risk

The genetic component of stroke has been carefully studied and is widely accepted [6, 21]. In large meta-analyses, FHS increased the risk of stroke by 30–44% [6]. In the current study, FHS increased the risk of stroke by 29% with further adjustment for Model 2, and by 18% with further adjustment for Model 3. A previous study also found that people with a positive FHS were more likely to have hypertension [22]. Hypertension is the most powerful risk factor for stroke and may thus account for an important component of the heritability of stroke [23, 24]. Both hypertension and stroke co-aggregate in families [25]. Hypertension may be one of the reasons for the hereditary nature of stroke.

The Framingham Study [8] found that parental stroke was associated with a three-fold increased risk of stroke in offspring, which persisted after adjustment for conventional stroke risk factors. However, Brass et al. [26] and Siegerink et al. [27] found that FHS could not predict the future stroke risk. A case-control study in the RATIO [28] showed that FHS was not significantly associated with IS risk. However, the current study found that FHS increases the risk of IS.

Meschia et al. [29], MacClellan et al. [30], and Kondo et al. [25] showed that sibling history was more significantly positively related to stroke risk than parental history. However, Liao et al. [31] did not find the same results. The present study did not find that a sibling history was associated with a significant increase in IS risk. Some researches have shown that maternal history of stroke was far more important than paternal history [32]. There may also be several reasons or underlying mechanisms, such as genomic imprinting and maternal intrauterine environments [33]. However, a meta-analysis [34] reported that the conferred risk of stroke in offspring did not differ substantially between positive paternal and maternal histories of stroke. This finding is similar to that of the present study.

In the subgroup analyses, significant differences in the association between FHS and IS were observed across the strata of smoking status and alcohol consumption. The association between FHS and IS risk was stronger in participants who were current smokers and current drinkers. These results are consistent with previous researches [35]. Stratified by age, the positive association between FHS and IS risk was slightly more pronounced among participants aged 30-60 years than among those aged >60 years (*P*_interaction_ = 0.004). A possible explanation is that the relative importance of FHS and genetic variables may decrease with increasing age, as other life-related factors take precedence [22].

### The association of WC and IS risk

WC is a widely used measurement of abdominal adiposity in epidemiology [36, 37], which might be a better indicator of accumulation of visceral fat and an adverse metabolic profile than BMI and the waist-to-hip ratio [38]. Many studies have found that excessive WC is associated with an increased risk of IS [39]. A previous study found that WC increased the risk of stroke by 28% [40]. The present study found that WC increased the risk of stroke by 78%. Visceral fat is associated with cardiovascular risk factors [41]. In the present study, the association between WC and incident IS was partially explained by this fact, because after adjustment for the BP and RPG in Model 3, the association was attenuated.

Abdominal adiposity is associated with well-established risk factors for stroke in general, including hypertension, dyslipidemia, and diabetes [42]. A Japanese study [43] showed a 22% increased risk of hypertension, 81% increased risk of dyslipidemia and 35% increased risk of diabetes with abdominal obesity. Stratified by age, the positive association between WC and IS risk was more pronounced among participants aged 30-60 years than among those aged ⩾ 60 years (*P*_interaction_ < 0.001). The additional increase in WC may be an important component of IS risk in younger adults. Stratified by sex, the positive association between WC and IS risk was more pronounced among females than males (*P*_interaction_ < 0.001). The explanation for sex differences may be found in the dietary, physical activity behavioral, and biological makeup of male and female differences. Females tend to have a higher body fat percentage than males, even in the same WC [44]. Abdominal adiposity is believed to be an important contributor to IS development. This is probably because visceral fat was considered as an endocrine organ with metabolic activity and secretion of pro-inflammatory cytokines obesity [45].

### The interaction of FHS and WC with IS risk

An association of FHS and WC with IS risk is lacking among the Chinese population. The interplay between genetic and environmental factors often makes it difficult to rely on particular genetic identification to determine the genetic risk of an individual’s cardiovascular disease [46]. We found a particularly significant interaction of FHS and WC with IS. The exact mechanism underlying the combined effect of FHS and WC on IS risk is not completely understood.

According to a case-control study of 6,000 participants, correctable risk factors are responsible for 90 % of stroke risk [47]. The effect of FHS on the risk of IS could reflect either genetic predisposition or shared lifestyle, both of which are not easy to modify in a short period. Thus, it is of great importance for individuals with FHS to decrease their WC by changing unhealthy lifestyles to reduce IS risk. Self-reported FHS information can also be used to personalize health messages and may be more effective than standardized health messages in motivating people to adopt and maintain a healthy lifestyle.

### Strengths and Limitations

The strengths of our study include the prospective cohort design, the large sample size, well-designed questionnaire, and systematic and uniform data collection and management. Height, weight, and WC were measured by trained and qualified investigators, using a uniform measurement protocol. This cohort had a low loss to follow-up and was a stable population. Data were collected with strict quality control and important covariates were measured and controlled for in the analysis. This study provided more credible evidence than case-control or cross-sectional studies.

However, this study has some limitations. First, the age at onset of first-degree relatives was not obtained from the questionnaire. Second, family history data were obtained based on self-report, which can lead to some degree of misclassification bias and recall biases, affecting the accuracy of the information. However, previous studies have reported that self-reported family history provides reliable stroke data [48]. Third, the study used RPG instead of fasting plasma glucose, which may have resulted in measurement error. However, a previous study on CKB showed that RPG is a good predictor of the risk of cardiovascular disease[36]. Finally, indices of adiposity were measured once at the baseline, therefore, the evaluation of changes in body size was not possible.

## Conclusion

In summary, we found that WC accentuated the impact of family history on IS. Our findings emphasize the importance of counselling family members regarding maintaining a recommended WC, collecting family history as a screening tool for individuals with increased disease risk, and considering gene-environment interactions in the prevention of IS.

## Figures and Tables

**Table 1:** The baseline characters of 33,355 participants according to family history of stroke and waist circumference.

Characteristic	All participants	FHS		WC (cm)	
		No	Yes	⩾85 (male)	≥85 (male)
				<80 (female)	⩾80 (female)
Percentage of participants (%)	100.00	84.33	15.67	37.37	62.63
Age (yr)	50.09±9.88	49.81±10.01	51.62±8.98	46.52±8.52	52.22±10.02
Female (%)	55.31	55.07	56.59	55.49	55.20
Education level (%)					
Primary school and below	21.68	21.65	22.04	10.65	28.26
Junior high school	41.91	41.45	44.40	45.18	39.96
High school and above	36.41	36.94	33.56	44.17	31.78
Occupation (%)					
Worker	42.73	43.69	37.61	57.34	34.02
Administration/Professional	9.87	10.19	8.15	12.11	8.54
Sales service staff/private	3.57	3.65	3.12	3.80	3.43
Housework	5.43	5.42	5.45	2.47	7.20
Retired, unemployed, laid-off, others	38.40	37.04	45.67	24.27	46.82
Annual Household income (yuan, %)					
<20000	39.17	38.56	42.45	34.61	41.89
20000-35000	44.04	44.68	40.62	47.90	41.74
⩾35000	16.79	16.76	16.93	17.49	16.37
Currently married (%)	92.92	93.02	92.37	94.19	92.15
Currently regular drinker (%)	64.71	64.20	67.50	71.23	60.83
Currently regular smoker (%)	28.84	28.62	30.07	31.27	27.40
Average daily consumption (%)					
Red meat	61.89	61.36	64.74	64.37	60.41
Fresh vegetable	98.11	97.97	98.81	98.11	98.10
Fresh fruit	54.19	54.30	53.61	56.05	53.08
Dairy products	33.58	32.99	36.77	37.88	31.02
Total physical activity (MET-h/d)	18.60±11.48	18.80±11.54	17.49±11.10	21.81±11.40	16.68±11.09
BMI (kg/m^2^)	25.56±3.45	25.50±3.45	25.91±3.44	22.78±2.15	27.23±2.97
SBP (mmHg)	131.35±20.78	130.86±20.61	133.95±21.51	123.64±17.95	135.94±21.00
RPG * (mmol/L)	6.29±2.60	6.26±2.55	6.43±2.81	5.81±1.95	6.57±2.88

Quantitative Values are presented as mean ± standard deviation or categorical values are presented as %; *P*-value refers to the comparison between participants with and without FHS, normal WC, and excessive WC. For each quantitative variable, the *P*-value is obtained by the method of Student’s t-test; for each categorical variable, the *P*-value is obtained by the Pearson’s x^2^ test.

* Data missing for 422 participants.

FHS, family history of stroke; WC, waist circumference; MET, metabolic equivalent of task; BMI, body-mass index; SBP, systolic blood pressure; RPG, random plasma glucose.

**Table 2:** Family history of stroke, waist circumference and risk of ischemic stroke.

	FHS			WC (cm)	
	No	Yes		<85 (male)	≥80 (female)
			<80 (female)	≥85 (male)
Overall				
No.of participants	28,128	5,227	12,466	20,889
No.of cases	866	227	186	907
PYs	253,713.83	48,295.05	116,004.89	186,003.99
Cases/PYs (/1000)	3.41	4.70	1.60	7.82
HR (95%CI)				
Model 1	1.00	1.28 (1.10,1.48)	1.00	1.82 (1.55,2.14)
Model 2	1.00	1.29 (1.12,1.50)	1.00	1.78 (1.51,2.10)
Model 3	1.00	1.18 (1.02,1.38)	1.00	1.42 (1.20,1.68)
Men				
No.of participants	12,639	2,269	5,549	9,359
No.of cases	405	125	118	412
PYs	115,531.51	21,094.02	52,232.36	84,393.16
Cases/PYs (/1000)	3.51	5.93	2.26	4.88
HR (95%CI)				
Model 1	1.00	1.54 (1.26,1.88)	1.00	1.68 (1.36,2.06)
Model 2	1.00	1.55 (1.27,1.90)	1.00	1.72 (1.40,2.12)
Model 3	1.00	1.37 (1.12,1.69)	1.00	1.38 (1.11,1.71)
Women				
No.of participants	15,489	2,958	6,917	11,530
No.of cases	461	102	68	495
PYs	138,182.32	27,201.03	63,772.52	101,610.83
Cases/PYs (/1000)	3.34	3.75	1.07	4.87
HR (95%CI)				
Model 1	1.00	1.06 (0.85,1.31)	1.00	2.04 (1.57,2.66)
Model 2	1.00	1.06 (0.86,1.32)	1.00	1.83 (1.40,2.39)
Model 3	1.00	1.00 (0.80,1.25)	1.00	1.48 (1.12,1.94)

FHS, family history of stroke; WC, waist circumference; PYs, person-years; HR, hazard ratio; CI, confidence interval.

## Data Availability

Details of how to access China Kadoorie Biobank data and details of the data release schedule are available from www.ckbiobank.org/site/Data+Access.

## Abbreviations

CKB: China Kadoorie Biobank
DSPs: Disease Surveillance Points System
FHS: family history of stroke
WC: waist circumference
IS: ischemic stroke
SBP: systolic blood pressure
DBP: diastolic blood pressure
BMI: body-mass index
RPG: random plasma glucose
MET: metabolic equivalent of task
HR: hazard ratio
CI: confidence interval
PYs: person-years
AP: attributable proportion due to interaction
RERI: elative excess risk due to interaction
SI: synergy index
RCS: restricted cubic splines
ICD-10: International Classification of Diseases, 10th revision
